# Investigation of photophysical properties and potential biological applications of substituted tris(polypyridyl)ruthenium(II) complexes

**DOI:** 10.3389/fchem.2025.1491598

**Published:** 2025-02-03

**Authors:** T. Sumitha Celin, G. Allen Gnana Raj, T. S. Prathima, M. M. Balamurali

**Affiliations:** ^1^ Department of Chemistry and Research Centre, Scott Christian College (Autonomous), Nagercoil, Tamilnadu, India; ^2^ Department of Chemistry, School of Advanced Sciences, Vellore Institute of Technology, Chennai, Tamilnadu, India

**Keywords:** tris(polypyridyl)ruthenium(II) complexes, binding studies, anticancer, antioxidant, antimicrobial

## Abstract

The photophysical properties of tris(polypyridyl)ruthenium(II) complex [Ru(dmbpy)_3_]^2+^ [dmbpy = 4,4′-dimethyl-2,2′-bipyridine] were investigated and compared with [Ru(bpy)_3_]^2+^ following both experimental and computational approaches. The variations in the electronic properties of the complex in the ground and excited states were determined by density functional theory (DFT) methods, and their effects on the anticancer, antioxidant, and antimicrobial activities were also evaluated by molecular docking and dynamic simulation studies. The potential of these complexes to serve as bioanalytes was investigated by their ability to bind with quinones, the well-known electron mediators in numerous light-driven reactions. Following the above, the anticancer properties were evaluated against breast cancer-related proteins. The results revealed that the complex possesses comparable anticancer and antioxidant potential to that of [Ru(bpy)_3_]^2+^. The physical, electronic, and biological properties of this complex depend on the nature of the ligands and the medium of investigation. Herein, the potential applications of [Ru(bpy)_3_]^2+^ in clinical diagnostics as antioxidants and therapeutic agents were evaluated.

## Graphical Abstract

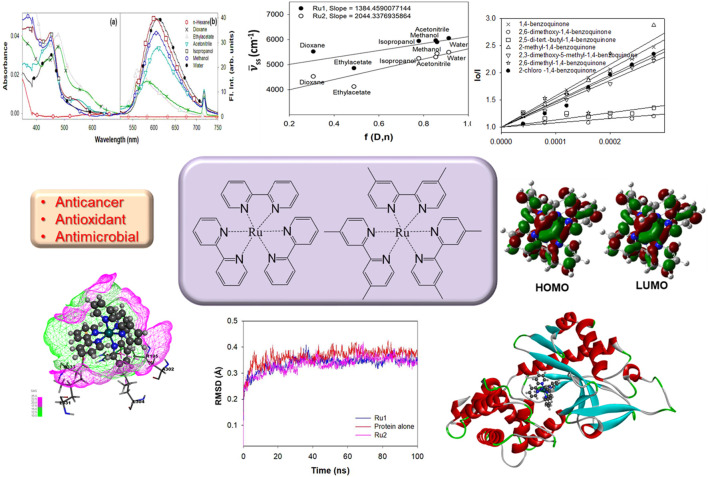

## Introduction

Heterocyclic compounds are an important class of organic molecules that have been utilized extensively in the field of medicinal chemistry (Abdul Rahman et al., 2023; Chahal et al., 2023; Kaur et al., 2023; Pal et al., 2024; Ravindar et al., 2024; Vijayakumar et al., 2023). Several fused heterocyclic compounds were well-recognized as biomedicines (Chen et al., 2016; Ghashghaei et al., 2018; Mi et al., 2021; Min et al., 2019), bioactive lead molecules (Chatterjee et al., 2023; Mandal et al., 2012; Matlock et al., 2014; Mekheimer et al., 2020), molecular recognition units (Benabdallah et al., 2018; Canard et al., 2016; Naseer et al., 2011; Neal et al., 2019), *etc*., in the process of drug discovery. The heterocyclic rings are present in the backbone of most of the available drugs and also in numerous bio-macromolecules like nucleic acids, proteins, vitamins, antibiotics, *etc*. They are also found abundantly in various natural products, agrochemicals, and pharmaceuticals. Despite these proven applications, these heterocycles are still in the limelight of research, and in the past few decades, these heterocycles have been investigated widely in drug discovery (Cho et al., 2023; Ogunnupebi et al., 2023; Pelliccia et al., 2023).

Several transition metal complexes belong to a class of chemotherapeutics with potent antitumor and antiviral pharmaceutical properties (Forooghi et al., 2024; Garcia-Garcia et al., 2024; Jagathesan and Roy, 2024; Noakes et al., 2024). To date, cisplatin and its analogs are considered the most effective antitumor drugs. However, the application of cisplatin as an anticancer therapeutic is limited by the developed resistance of primary tumors, water solubility issues, associated adverse side effects, and high toxicity (Boulikas and Vougiouka, 2003; Popolin and Cominetti, 2017; Sedletska et al., 2005; Spirina et al., 2020). Although numerous alternate drugs are available (Abu-Surrah and Kettunen, 2006), cisplatin is still used as a combination drug in the treatment of numerous cancers (Hsu et al., 2023; Raina et al., 2023). The above limitations have paved the way for the search for other transition metal complexes with potent biological properties, broad-scope reactivity, and low systemic toxicity (Das et al., 2023; Rani et al., 2023; Sharma et al., 2023). Recent investigations on ruthenium complexes have projected them as potent alternatives to cisplatin. Investigations on ligand-dependent cytotoxicity, lipophilicity-induced cellular uptake, and anti-proliferation potential are underway.

In the past few decades, efforts have been made to investigate the potential of ruthenium complexes as the most promising anticancer therapeutics (Chen et al., 2022; Guo et al., 2024; Hildebrandt et al., 2022; Wang et al., 2023; Xiong et al., 2022). Their unique ligand exchange kinetics in an aqueous medium and low cytotoxicity play crucial roles in the observed anti-tumor activity and have hence been projected as attractive alternatives to platinum-based drugs.

To date, numerous ruthenium complexes have been reported as potent anticancer agents (Chen et al., 2022; Guo et al., 2024; Hildebrandt et al., 2022; Wang et al., 2023; Xiong et al., 2022). Recently, the lipophilicity of several tris(polypyridyl)ruthenium(II) complexes possessing bipyridyl-derived ligands was evaluated for their anticancer potentials (Elgar et al., 2023). The studies have revealed that most of the lipophilic compounds are known to exhibit cytotoxicity by adhering themselves to the plasma membrane, compared to compounds with less lipophilicity that can penetrate and accumulate in various sub-cellular organelles in the cytoplasm (Batchelor et al., 2017; Li et al., 2022; Lv et al., 2015; Thangavel et al., 2024). The therapeutic potential of these complexes to target nucleic acids is scarcely reported in the literature (Gorner et al., 1988; Hu et al., 2022; Lopes-Costa et al., 2009; Tossi and Gorner, 1993), as these complexes were never reported to target and accumulate in the cell nucleus. Several studies have revealed ruthenium complexes as potential diagnostic and therapeutic agents for numerous biomedical applications.

The potential applications of ruthenium complexes in various fields of chemistry, including photochemistry, electrochemistry, photo-catalysis, electron/energy transfer reactions, luminescent sensors, *etc*., have been reported (Behroozi et al., 2024). A highly sensitive electro-generated chemiluminescent sensor with tris(2,2′-bipyridyl)ruthenium(II) on a graphene-titania-Nafion composite film was reported to exhibit good response for nicotinamide adenine dinucleotide reduced form (NADH) with a detection limit of 0.4 μM, thus serving as a promising scaffold for the development of dehydrogenase-based biosensors (Lee et al., 2022). Similarly, an array of tris(polypyridyl)ruthenium(II) protein surface mimetics was recently reported to sense and differentiate proteins possessing important therapeutic targets *via* linear discriminant analysis (Hewitt and Wilson, 2017). The tris(polypyridyl)ruthenium(II) complexes are known for their distinct chemical stability in the ground and lowest excited singlet states. Their stabilities extend to their reduced and oxidized forms. This unique property makes them an efficient molecule for various electron transfer reactions. For the same reason, investigations on the synthesis and application properties of various ruthenium complexes are still a top research priority.

**SCHEME 1:**
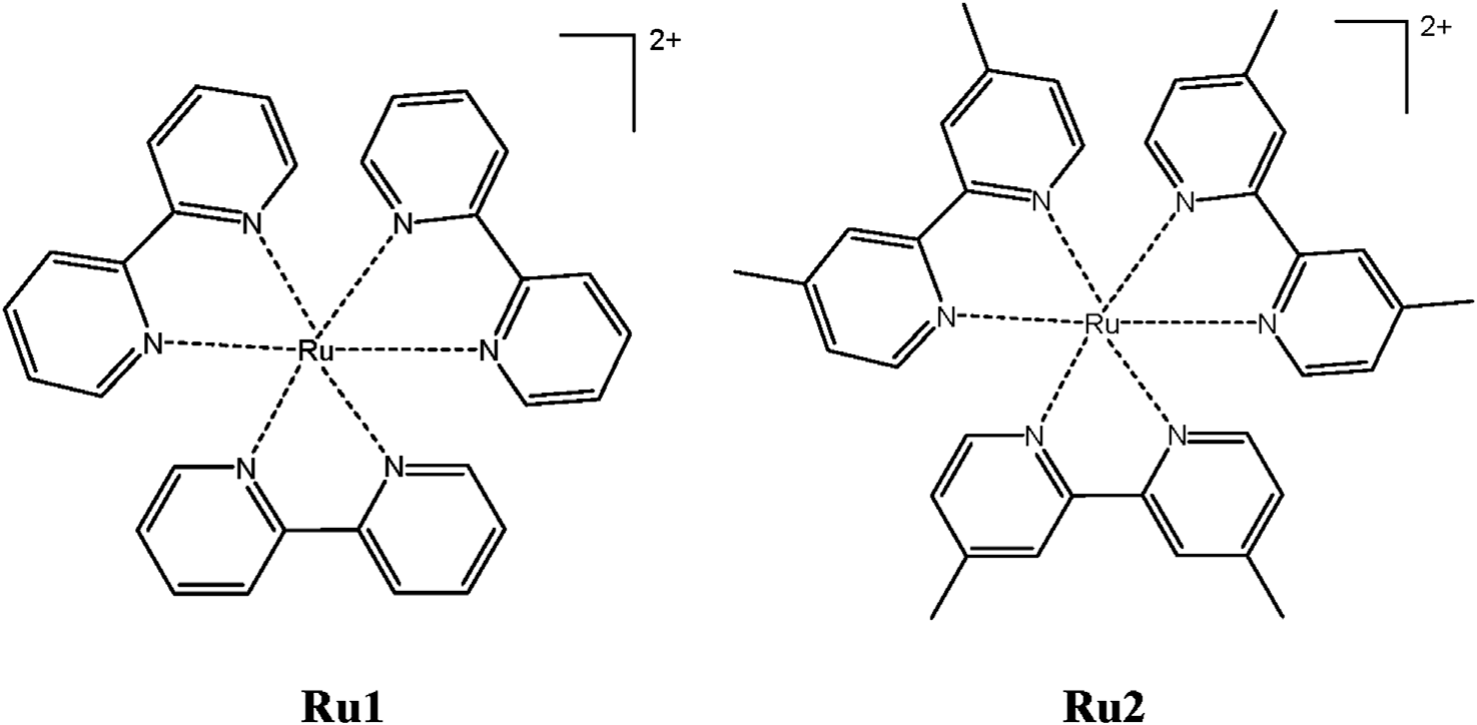
Chemical structures of tris(polypyridyl)ruthenium(II) complexes.

With all the above unique features, the present investigation focuses on the photophysical, electronic, and biological properties of [Ru(dmbpy)_3_]^2+^ (**Ru2**) [bpy = 2,2′-bipyridine and dmbpy = 4,4′-dimethyl-2,2′-bipyridine] and comparing those properties with the well-known complex tris(polypyridyl)ruthenium(II) complexes [Ru(bpy)_3_]^2+^ (**Ru1**) (Scheme 1).

## Materials and methods

All the chemicals used in this study were obtained from Sigma-Aldrich and were used as received without further purification. Solvents used for the study, including n-hexane, dioxane, ethyl acetate, acetonitrile, isopropanol, and methanol, were procured from Merck. All the solvents were either high-performance liquid chromatography (HPLC) or spectroscopy grade and were used as received. Distilled water was used to prepare the aqueous solutions.

The procedure employed to synthesize the ruthenium complexes was reported elsewhere (Basudeb Saha and Stanbury, 2000; Swarnalatha et al., 2011), and the relevant data are given in Supplementary Material. The stock solutions of ruthenium complexes for photophysical and electronic studies were prepared in DMSO at a concentration of ∼10^−3^M.

### Binding energy calculations

To understand the nature and mechanism of interaction between the protein and ligand complexes, docking investigations were performed employing the Schrödinger interfaced GLIDE module, and the binding energies were computed. A grid-based ligand docking algorithm was utilized to calculate the accurate protein–ligand interactions and the associated orientations. The previously reported binding cavity of 4WOY protein was used to assign the above grid. By following all the above strategical assignments and parameters, the proposed tris(polypyridyl)ruthenium(II) complexes were evaluated for their ability to interact effectively with the binding cavity of 4WOY. All conclusions were based on the associated binding energies and docking scores. The BIOVIA DSV tool was used to visualize the binding interactions in their docked conformations.

With the above-docked structures, further refinement was done by following MM-GBSA procedures with a flexible residue distance of 5 Å to evaluate the overall binding free energy. To eliminate the false positives, rescoring and validating the above-docked postures were carried out. Following the standard precision (SP) G-Score, the binding poses of each small molecule were ranked accordingly. The prime MM-GBSA module of the Schrödinger suite was employed to calculate the overall binding free energies with the help of pose viewer files obtained from the above GLIDE docking evaluations. The factors contributing to the binding free energy include interaction energies like *van der* Waal’s, electrostatic, entropy terms, polar and nonpolar contributions of the ligand molecules, *etc*.

### Molecular dynamic simulations

The molecular dynamic simulations were carried out with the help of the DESMOND tool of the Schrödinger suite. The protein–ligand complexes were placed in an orthorhombic box with a simple point charge (SPC) solvent model using the system builder panel. Further neutralization of the solvated system was done by adding counter ions to a physiological salt (NaCl) concentration of 150 mM. The OPLS4 force field was utilized for the simulation, and the time period was fixed to 100 ns along with the constant-temperature, constant-pressure ensemble (NPT) ensemble class at 300 K and 1.013 bar atmospheric pressure.

## Results and discussion

### Evaluation of the photophysical properties of ruthenium complexes

It is well known that the electronic properties of metal complexes play crucial roles in tuning their interactions with biomolecules. In particular, the charge transfer between the metal and ligands is of great interest while exploring the bio-applications of metal complexes. Herein, the photophysical properties of the **Ru2** complex were investigated under different environmental conditions following their electronic absorption and fluorescence emission and excitation spectra in different solvents of varying polarity and protic nature and compared with those of **Ru1**.

**FIGURE 1:**
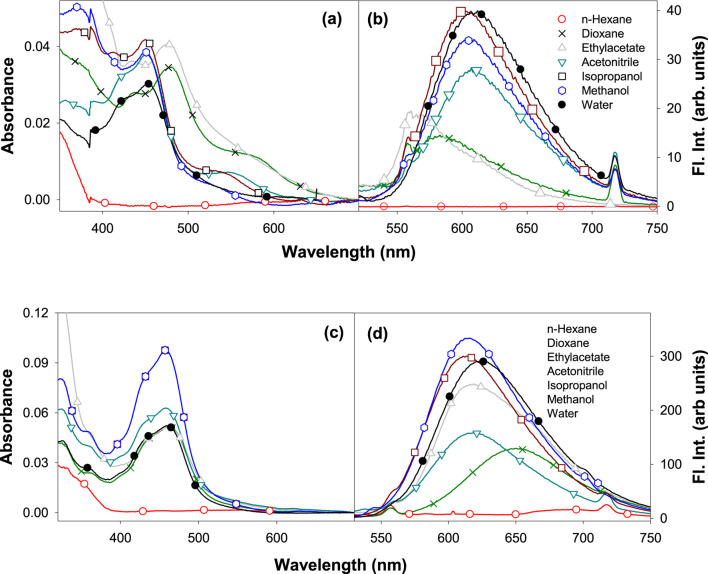
Electronic absorption of **(A) Ru1** and **(C) Ru2** and the fluorescence emission spectra (λ_ex_ = 480 nm) of **(B) Ru1** and **(D) Ru2** in different solvents.

The ground state electronic properties of **Ru2** were investigated from their absorption spectra recorded in the range of 250 nm–750 nm in different solvents: n-hexane, dioxane, ethyl acetate, acetonitrile, isopropanol, methanol, and water (Charles et al., 2019). The concentration of all the samples was maintained so that the absorbance values did not exceed 0.1 absorbance units. The absorption spectra of **Ru1** and **Ru2** in different solvents are depicted in Figures 1A, C, respectively. Further evaluations were made based on the long wavelength absorption band of the spectrum in respective solvents. In all the cases, as the polar and protic nature increases, a slight blue shift was observed in the spectrum. A weak band in the long wavelength tail region indicates the origin of the d-d transition. The possibility for charge transfer transition was neglected as the observed molar extinction coefficient was very low. Moreover, the molar absorptivity values revealed that the nature of transition involved is π-π* in nature, which could have originated from the ligand transitions.

Moreover, the presence of multiple species in the ground state can be ruled out as the full width at half maxima (FWHM) is ∼2,500 cm^−1^. The structure observed in the spectrum could be due to the transitions arising from the isoelectronic states of planar bipyridyl moieties of the complexes. The spectrum could not be recorded in water, possibly due to the poor solubility of complexes or to the formation of other ionic species with negligible molar extinction coefficients at pH ∼ 7. The values of band maxima and molar extinction coefficient for all the complexes in different solvents are given in Table 1. The band at approximately 450 nm could be due to the metal–ligand charge transfer (MLCT) dπ-π* transition, while the band at approximately 350 nm could be due to the d-d transition. The band observed at approximately 285 nm corresponds to the π-π* ligand-centered (LC) transition arising from the bipyridyl ligands.

**TABLE 1:** Electronic spectral properties of **Ru1** and **Ru2** complexes in different solvents.

Sample	Solvent	$\lambda_{\max}^{ab}$ (log $\varepsilon_{\max}$ )	$\lambda_{\max}^{fl}$
Ru1	n-Hexane	—	—
	Dioxane	437, **480** (*3.54*), and 560 (*sh*)	**583**
	Ethyl acetate	437, **478** (*3.61*), and 560 (*sh*)	**598**
	Acetonitrile	426 (*sh*), **451** (*3.58*), and 546 (*sh*)	**608**
	Isopropanol	410, **450** (*3.6*2), and 525 (*sh*)	**604**
	Methanol	410 (*sh*), **449** (*3.59*), and 520 (*sh*)	**604**
	Water (pH ∼7)	430 (*sh*), **452** (*3.48*), and 510 (*sh*)	**612**
Ru2	n-Hexane	—	**695**
	Dioxane	359, 407 (*sh*), 439 (*sh*), and **460** (3.71)	**650**
	Ethylacetate	359 (*sh*), 439 (*sh*), and **460** (3.71)	**618**, 647 (*sh*)
	Acetonitrile	359 (*sh*), 410 (*sh*), 439 (sh), and **457** (3.80)	**617**
	Isopropanol	359 (*sh*), 408 (*sh*), 437 (*sh*), and **456** (3.85)	**612**
	Methanol	359 (*sh*), 400 (*sh*), 432 (*sh*), and **455** (3.99)	**613**
	Water (pH ∼7)	359 (*sh*), 400 (*sh*), 432 (*sh*), and **455** (3.71)	**624**

*Bold values indicate the prominent long wavelength absorption and emission bands.

The electronic properties of complexes in their excited state were evaluated by following their fluorescence emission spectra in different solvents of varying polarity and protic nature. The presence of a single species in the excited state was revealed from the identical spectrum obtained at different excitation wavelengths (λ_ex_: 350 nm, 430 nm, and 480 nm) chosen in the region with a significant molar extinction coefficient of the absorption spectrum. Moreover, the FWHM values ∼3,400 cm^−1^ also reveal the same. The values of the Stokes shift are given in Table 2 and further reveal that no significant excited state reactions like excimer or exciplex formation occur upon excitation. The respective fluorescence spectra in different solvents are depicted in Figures 1B, D. The spectrum could not be recorded in n-hexane due to its poor solubility. Moreover, in weakly polar solvents, the spectra were slightly structured, indicating a more planar geometry that decreases as the solvent polarity and protic nature increase. In the case of water, the trend was not observed. This could be due to the formation of other ionic species at pH ∼7. Further investigation on prototropic properties would shed light on the observed emission spectra in water.

**TABLE 2:** Values of Stokes shifts computed from the electronic absorption and fluorescence emission spectra in different solvents.

Solvent	Stokes shift ( ${\bar{\nu }}_{ss}$ cm^−1^)	
	Ru1	Ru2
n-Hexane	—	—
Dioxane	5,516	4,522
Ethyl acetate	4,853	4,117
Acetonitrile	5,894	5,435
Isopropanol	5,936	5,243
Methanol	5,959	5,298
Water (pH ∼7)	6,049	5,488

Unlike the absorption spectra, the emission spectra of **Ru2** were blue shifted (∼82 nm) compared to **Ru1**, which was slightly red shifted (∼21 nm) with increasing polarity and protic nature of solvents. This indicates that the dipole moment of the complexes in the excited state is either lower or has negligible changes upon excitation in the case of **Ru2**, with negligible solvent-induced dipolar interactions, while with **Ru1**, the dipolar interactions were significant. The observed structure in the spectrum reveals the occurrence of vibrational transitions that are not from different species, as the excitation spectrum around the region of emission wavelengths did not change at different emission wavelengths. This also indicates the excited state species is photo-stable and does not degrade or undergo a reaction like excimer/exciplex formation throughout the singlet state lifetime.

### Computational evaluations on the solvatochromic effects of ruthenium complexes

**FIGURE 2:**
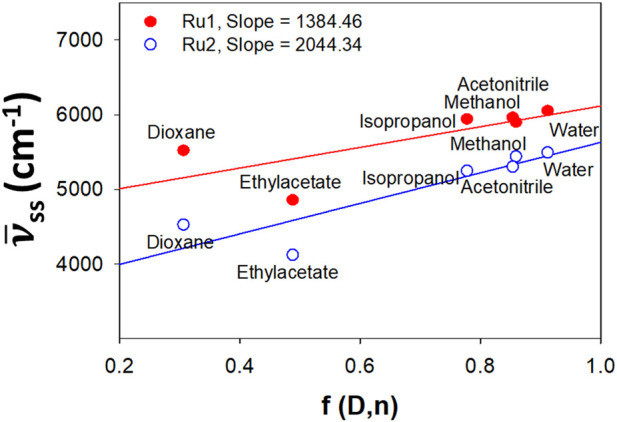
Lippert plot depicting the effect of solvatochromism induced by general solvent effects for **Ru2** compared to **Ru1**.

Investigations into the solvatochromic properties of **Ru2** were carried out and compared with **Ru1** *via* the plot of Stokes shift vs. Lippert’s solvent polarity parameters (Figure 2). The Lippert–Mataga (Equation 1) takes into account only the general solvent effects and ignores specific solvent effects like hydrogen bonding. The change in dipole moment (∆µ) can be estimated from the slope of the plot, while the intercept reveals the Stokes shift in the absence of any solvent. The plot reveals linearity irrespective of the solvents’ polarity or protic nature, indicating that the specific solvent effects do not influence the electronic spectral properties.

$$\gamma_{\text{ss}}=\left[2{\left({µ}_{e}-{µ}_{g}\right)}^{2}/{\text{hca}}^{3}\right]∆f+\gamma_{\text{ss}}^{o},$$ (1)

where γ_ss_ and γ_ss_^o^ are the Stokes shift in the presence and absence of any solvent, respectively, and a is the Onsager cavity radius, which was taken as 40% of the maximum length of the complexes. ∆f is the orientation polarizability (Equation 2), which was calculated as

$$\Delta f=\left[\left(\varepsilon -1\right)\;/\;\left(2\varepsilon +1\right)\right]\;–\;\left[\left(n^{2}\;–\;1\right)\;/\;\left(2n^{2}+1\right)\right],$$ (2)

where ε is the dielectric constant of the solvent, and n is the refractive index of the same.

### Influence of quinones on the charge transfer properties of ruthenium complexes

The charge transfer properties and electronic charge delocalization in metal complexes determine their biological applications, as the interactions are mostly charge-centered and non-specific other than hydrogen bonding. Quinones are well-established electron transfer mediators in various photosystems, and this property has been utilized to develop numerous therapeutics. The electron transfer reactions between various quinone derivatives and electronically excited ruthenium complexes were reported in the literature (Digby et al., 2020; Nyland et al., 2010; Son et al., 2017; Zhang et al., 2024). Herein, the influence of various quinones on the excited states of **Ru2** was investigated and compared with that of **Ru1** by following the Stern–Volmer equation, which evaluates the nature of quenching associated with the excited molecules. The linearity observed in the plot indicates that the associated quenching was dynamic. The plot also indicates that there is no ground state complex formation between the ruthenium complexes and the quinones.

**FIGURE 3:**
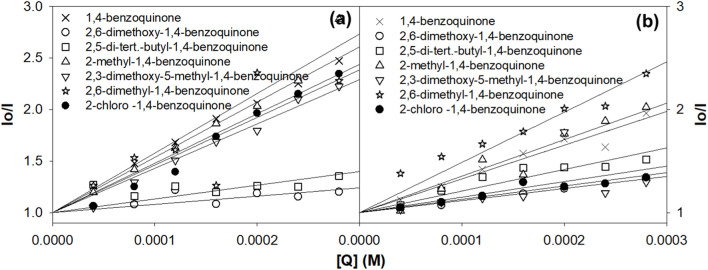
Evaluation of the Stern–Volmer quenching constant from the plot of concentration vs*.* I_0_/I. **(A)** **Ru1** and **(B)** **Ru2**.

The slope of the graph gives the Stern–Volmer quenching constant, and the same is given in Table 3. The observed quenching constant values were in nM concentrations. The Stern-Volmer (Equation 3) is given as

$$I_{0}\;/\;I=1+K_{\text{sv}}\;\left[Q\right],$$ (3)

where *I*_*0*_ and *I* indicate the fluorescence intensities in the absence and presence of quinones (quencher), respectively. *K*_*sv*_ is a linear Stern–Volmer quenching constant, and [Q] is the quencher concentration. The quenching constant can be calculated using the plot of (*I*_*0*_ */I*) *versus* [Q] (Figure 3).

**TABLE 3:** Values of the Stern–Volmer quenching constant for tris(polypyridyl)ruthenium(II) complexes.

Quinone	K_SV_ (nM^−1^)	
	Ru1	Ru2
1,4-Benzoquinone	5.35	3.27
2,6-Dimethoxy-1,4-benzoquinone	0.80	1.17
2,5-Di-tert-butyl-1,4-benzoquinone	1.33	2.09
2-Methyl-1,4-benzoquinone	5.77	3.54
2,3-Dimethoxy-5-methyl-1,4-benzoquinone	4.30	1.50
2,6-Dimethyl-1,4-benzoquinone	4.79	4.87
2-Chloro-1,4-benzoquinone	4.61	1.29

#### Evaluation of binding potential with quinones

The binding of the ruthenium complex **Ru2** with different concentrations of various quinones was evaluated from their absorption spectra and compared with that of **Ru1**. The binding constants (K_b_) of the complexes with various quinones were determined from the Benesi–Hildebrand equation (Equation 4).

$$\frac{1}{∆A}=\frac{1}{K_{b}}∆\varepsilon \;\left[H\right]+\frac{1}{∆\varepsilon }\;\left[Q\right],$$ (4)

where ΔA is the change in the absorption of the complex with various concentrations of quinones. The binding constant K_b_ was calculated from the slope and intercept of the linear plot of 1/ΔA versus 1/[Q], and the same is given in Table 4.

**TABLE 4:** Values of binding constant, K_b_ (M^−1^), for 1,-benzoquinone, 2,6-dimethyl-1,4-benzoquinone, and 2,5-dimethoxy-1,4-benzoquinone in an aqueous medium.

Quinone	Binding constant (K_b_) (M^−1^)	
	Ru1	Ru2
1,4-Benzoquinone	1.76 × 10^4^	2.74 × 10^4^
2,6-Dimethyl-1,4-benzoquinone	3.14 × 10^4^	5.41 × 10^4^
2,5-Dimethoxy-1,4-benzoquinone	3.34 × 10^4^	9.75 × 10^4^

The absorption spectral studies of ruthenium complexes show a steady increase in the metal-to-ligand charge transfer (MLCT) absorption maximums, with the incremental addition of various quinones. These results indicate the formation of a ground state complex. The addition of 1,4-benzoquinone to the complexes increases the absorbance at 290 nm and 448 nm, showing the binding of quinone to the complexes. All the quinones show a weak absorption near the MLCT absorption band of the complexes. The binding of quinones was revealed from the LC and MLCT absorption peaks of the complexes in their ground state (Abu-Surrah and Kettunen, 2006). The K_b_ values of the complexes with the quinones 1,4-benzoquinone, 2,6-dimethyl-1,4-benzoquinone, and 2,5-dimethoxy-1,4-benzoquinone are tabulated in Table 4.

**FIGURE 4:**
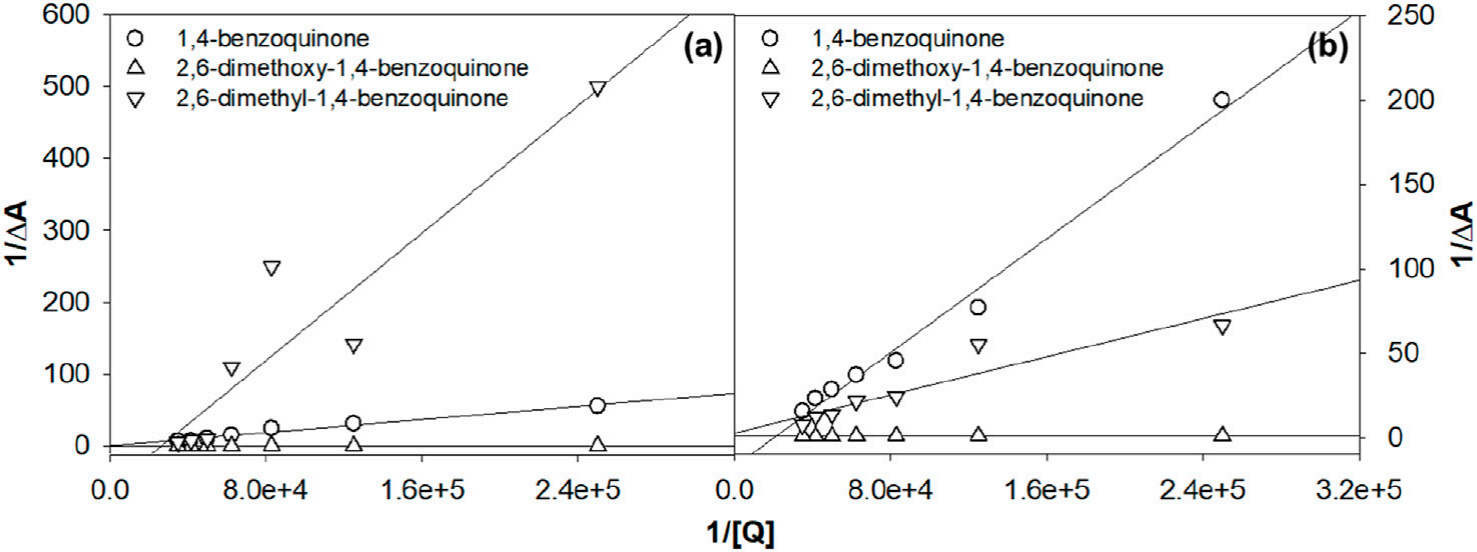
Benesi–Hildebrand plot for the tris(polypyridyl)ruthenium(II) complexes **(A) Ru1** and **(B) Ru2**.

The binding constant of compound **Ru1** for 1,4-benzoquinone, 2,6-dimethyl-1,4-benzoquinone and 2,5-dimethoxy-1,4-benzoquinone was 1.748 × 10^4^ M^−1^, 3.138 × 10^4^ M^−1^, and 3.34 × 10^4^ M^−1^, respectively. In comparison, the **Ru2** complex displayed an increase in the intrinsic binding constant value of 2.729 × 10^4^ M^−1^, 5.41 × 10^4^ M^−1^, and 9.75 × 10^4^ M^−1^, respectively, for the same quinones. These findings demonstrate that when 1,4-benzoquinone is substituted with 2,5-dimethoxy-1,4-benzoquinone, the K_b_ value increases. In the case that the ligand of the ruthenium complex is switched from bipyridine to dimethyl bipyridine, the value of the binding constant is further increased. This could be due to the steric effect, which plays an important role in influencing the binding constant values. The Benesi–Hildebrand plot depicting the variation in the MLCT absorption band of the **Ru1** and **Ru2** complex upon increasing the concentrations of various benzoquinone is displayed in Figure 4.

### Computational evaluation of the electronic properties

**FIGURE 5:**
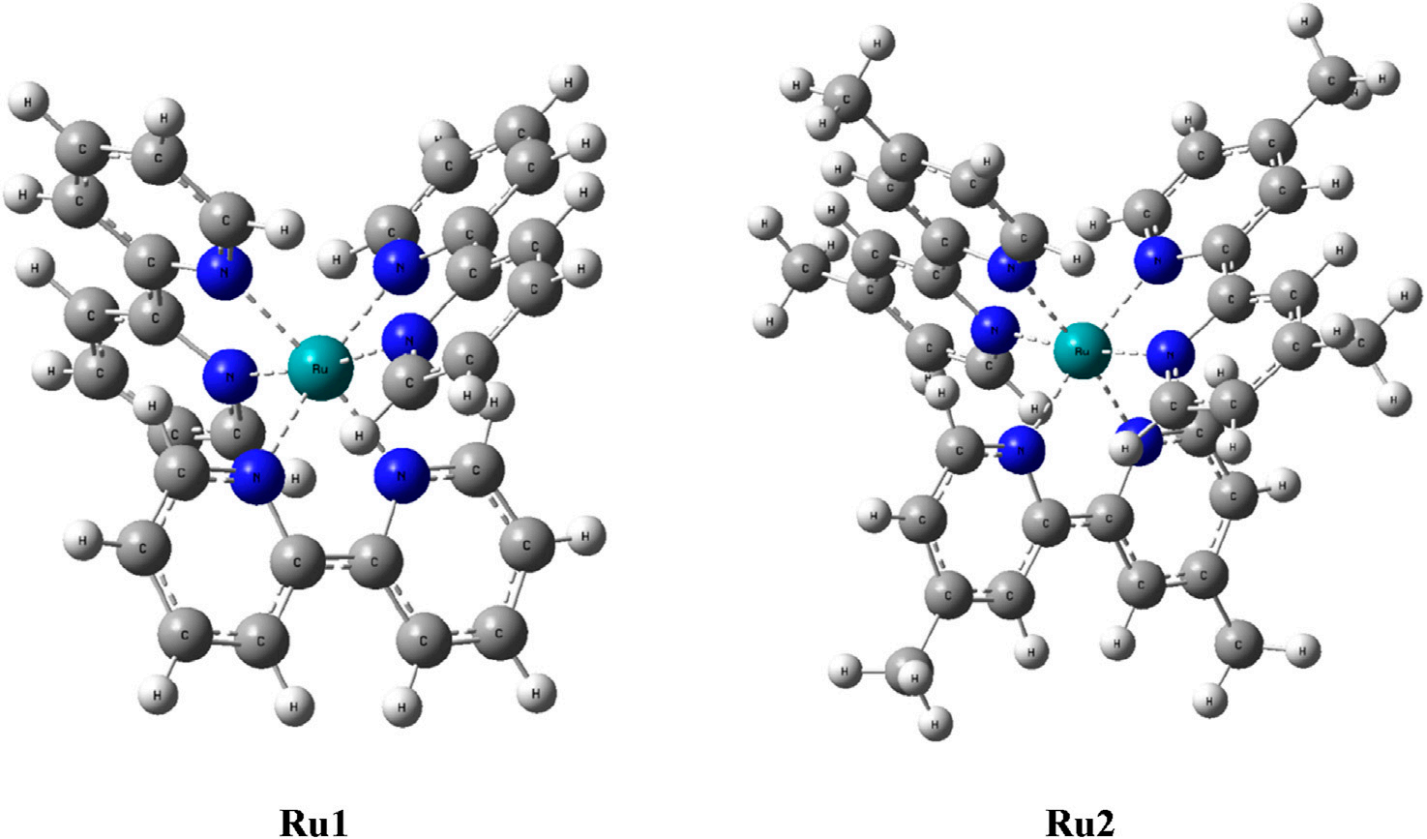
Geometry-optimized structures of tris(polypyridyl)ruthenium(II) complexes.

In order to evaluate the electronic properties of *tris(polypyridyl)ruthenium(II) complexes,* density functional theory calculations were carried out under isolated conditions in the absence of any solvents. The geometry of the complexes was optimized in their ground singlet states, and the corresponding coordinates are given in Supplementary Table S1. The geometries of energy-minimized structures are depicted in Figure 5. Geometry optimizations were not performed for the excited state to minimize the computational cost and time. Instead, a time-dependent DFT calculation was performed with vertical excitation possessing the identical ground state geometry. The relevant data are given in Table 5. The attainment of energy-minimized structures was ascertained by the absence of any negative frequencies at the end of the optimization procedures. Careful examination of the geometries revealed that the bipyridyl ligands coordinating the central ruthenium are aligned in an octahedral orientation for both complexes. The geometry-optimized structures are depicted in Figure 5.

**TABLE 5:** Computed parameters for the geometry-optimized structures.

	Ru1		Ru2	
Energy (eV)	−42991.01		−49409.55	
Dipole moment				
µg (D)	0.0024		0.0047	
µe (D)	0.0057		0.0087	
Charge density on heteroatoms	N_4_	−0.3366	N_4_	−0.3334
	N_8_	−0.3359	N_8_	−0.3333
	N_16_	−0.3363	N_16_	−0.3336
	N_20_	−0.3360	N_20_	−0.3337
	N_28_	−0.3364	N_28_	−0.3337
	N_32_	−0.3364	N_32_	−0.3338
	Ru_37_	0.9289	Ru_37_	0.9148
Excited-state transitions				
Excited state 1	573.08 nm (0.0002)		588.80 nm (0.0002)	
	H-1 → L (100%)		H-1 → L (98.2%)	
Excited state 2	489.52 nm (0.0664)		457.99 nm (0.1863)	
	H-3 → L (47.4%)		H-1 →L+2 (2.3%)	
	H-2 → L+1 (46.9%)		H → L+8 (92.3%)	
Excited state 3	446.87 nm (0.1420)		430.01 nm (0.0393) H → L+10 (93.6%)	
	H-1 → L+2 (3.1%)			
	H → L+9 (90.8%)			

The dipole moment computed for **Ru2** indicates that the complex was weakly polarized, similar to **Ru1** under isolated conditions, which could be the reason for the observed weak shift in the electronic spectra of both complexes in different solvents. The excitation energies were evaluated for various Franck–Condon transitions, and the energies associated with the first three excited singlet states are given in Table 5. The value of oscillator strength is given in parenthesis, while the contributing transitions, along with their percentage contribution for a given excited state, are also listed in Table 5.

**FIGURE 6:**
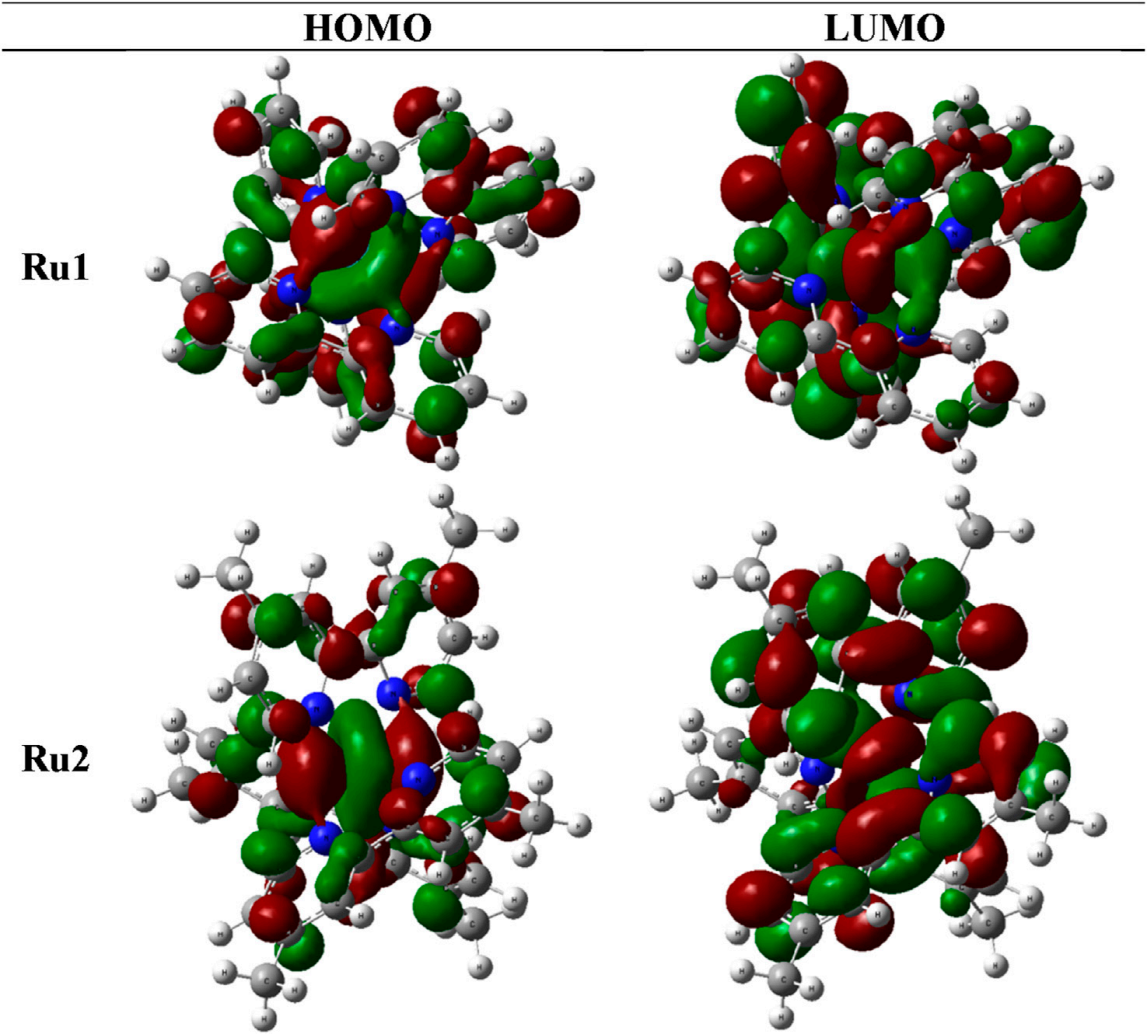
Charge distribution of **Ru1** and **Ru2** in their respective HOMOs and LUMOs.

The molecular orbitals and charge distribution in the HOMO and LUMO of **Ru1** and **Ru2** are shown in Figure 6. It could be seen that the charges were distributed throughout both **Ru1** and **Ru2**.

Further efforts to investigate the biological application of these complexes were attempted based on the information available from evaluating the electronic properties. The drug likeliness and biomolecular interaction potentials are greatly influenced by the structural and electronic properties, including planarity, charge densities, dipole moment, etc. Investigations like computational anticancer evaluation and experimental antioxidant and antimicrobial properties were performed for the complexes.

### Evaluation of anticancer potential

**FIGURE 7:**
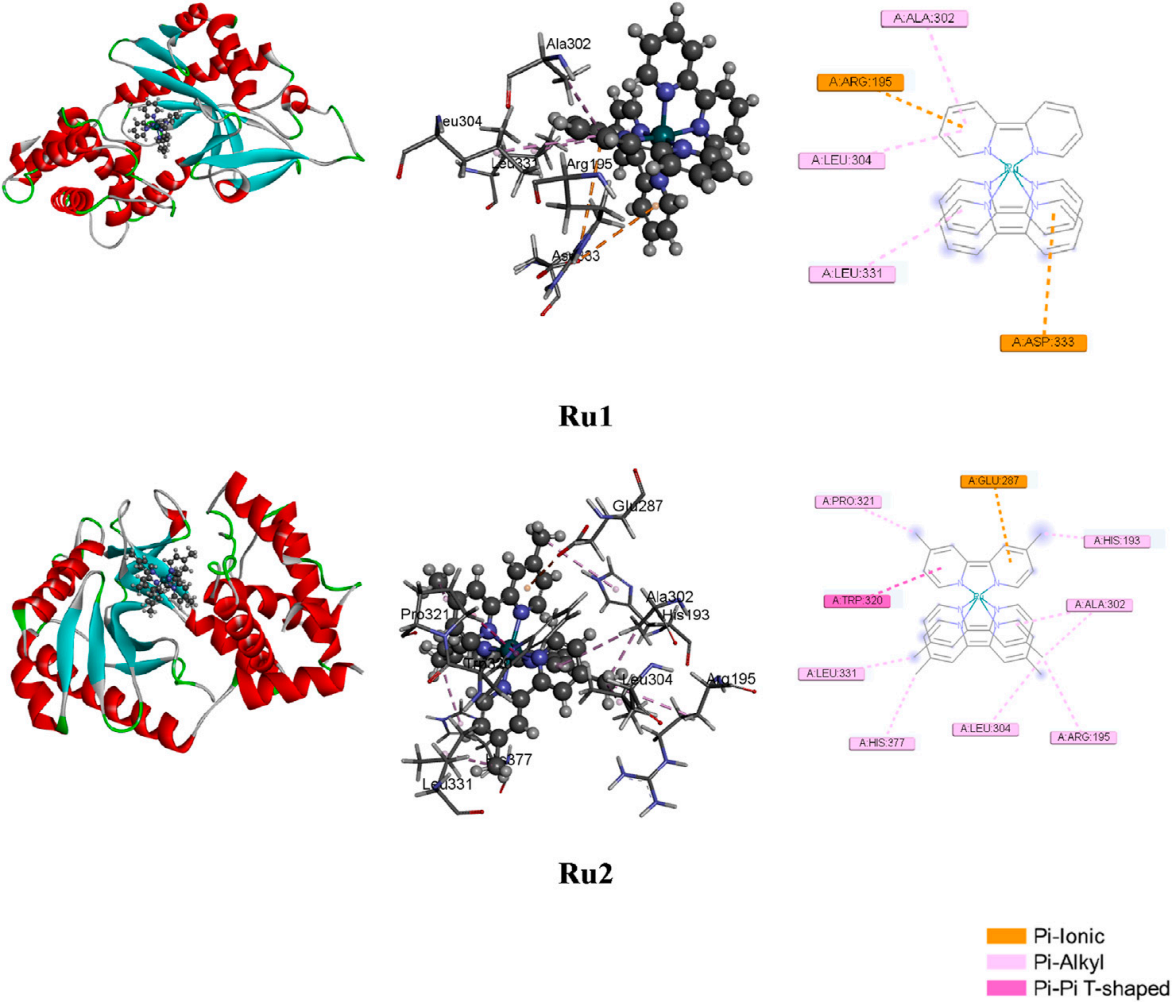
Docked postures of **Ru1** and **Ru2** with the 4WOY receptor protein of mitochondrial fission protein Drp1.

To investigate the biological significance of the reported ruthenium complexes, molecular docking investigations were carried out against the MiD49 protein. The latter not only acts as a receptor for the mitochondrial fission protein Drp1 but also plays significant roles in influencing cancer progression. The X-ray crystal structure of the protein (Pdb # 4WOY) was obtained from the Brookhaven Protein Data Bank. The BIOVIA Discovery Studio 2021 (DSV) client was employed to visualize the protein structure. In order to investigate the interaction potential of the ruthenium complexes with the above protein, docking investigations were carried out with the help of the GLIDE module of the Schrödinger suite, and the binding affinity of the ruthenium complexes was evaluated. The protein–ligand binding orientations were predicted accurately with the help of the grid-based ligand docking algorithm. Following the above strategic techniques, the influence of ligand binding to block the receptor site of MiD49 was analyzed. The ligand-docked structures were further investigated based on their binding free energies and docking scores. Geometry optimizations of the ruthenium complexes were performed following an energy minimization procedure employing DFT methods with LANL2DZ basis set and B3LYP functional using Gaussian 09. The free energies of binding were computed with the help of the MM/GBSA module of Schrödinger following Equation 5. The same module was used to re-score and validate the results obtained from docking calculations, and the false positives were eliminated.

$$\Delta G_{\text{bind}}=\Delta E_{\text{MM}}+\Delta G_{\text{solv}}+\Delta G_{\text{SA}},$$ (5)

where ΔE_MM_ denotes the energy difference between the protein–ligand complex and the ligand-free protein and liganded protein. Solvation energy, denoted as ΔG_solv_, is the energy difference between the protein–ligand complex and the total energy values combining the ligand-unbound and ligand-bound protein states. The surface area-associated free energy is represented as ΔG_SA_ and is the energy difference between the protein–ligand complex and the sum of energies associated with the surface area for ligand-free protein and ligand-bound protein. The evaluation of free energies of binding was performed by utilizing the reference and hit molecules obtained from the position viewer and as inferred from gliding docking investigations. Parameters like *van der Waal’*s interaction, electrostatic interactions, and entropy terms were also included in the evaluation of binding energies. The docked postures and the various interactions are depicted in Figure 7, and the list of interacting residues is given in Table 6.

**TABLE 6:** List of interacting residues influencing the binding to the 4WOY protein.

Ligand	Interacting residues
Ru1	R193, A302, L304, L331, and D333
Ru2	H193, R195, E287, A302, L304, W320, P321, L331, and H377

### Molecular dynamics simulations

In order to evaluate the dynamic nature of interactions between the protein and ligand complexes, molecular dynamic simulations were performed, and the various parameters, including root mean square distance (RMSD), root mean square fluctuation (RMSF), radius of gyration, and solvent accessible surface area, were investigated. The simulations were carried out with the DESMOND module of Schrödinger. The calculations were performed with the SPC water model for a simulation period of 100 ns with an NPT ensemble class at 300 K and 1.013 bar atmospheric pressure.

#### Influence of the interaction of ruthenium complexes on the RMSD trajectory of 4WOY

The variation in structural conformation of the protein backbone (4WOY) upon the approach of ligand (**Ru1** or **Ru2**) during the simulation period of 100 ns was evaluated by following its RMSD. Negligible deviation from its mean position indicates that no significant conformational changes were induced to the protein structure upon its interaction with the ligands. Thus, the stability of the protein–ligand complexes can be evaluated by following their respective RMSD trajectories.

**FIGURE 8:**
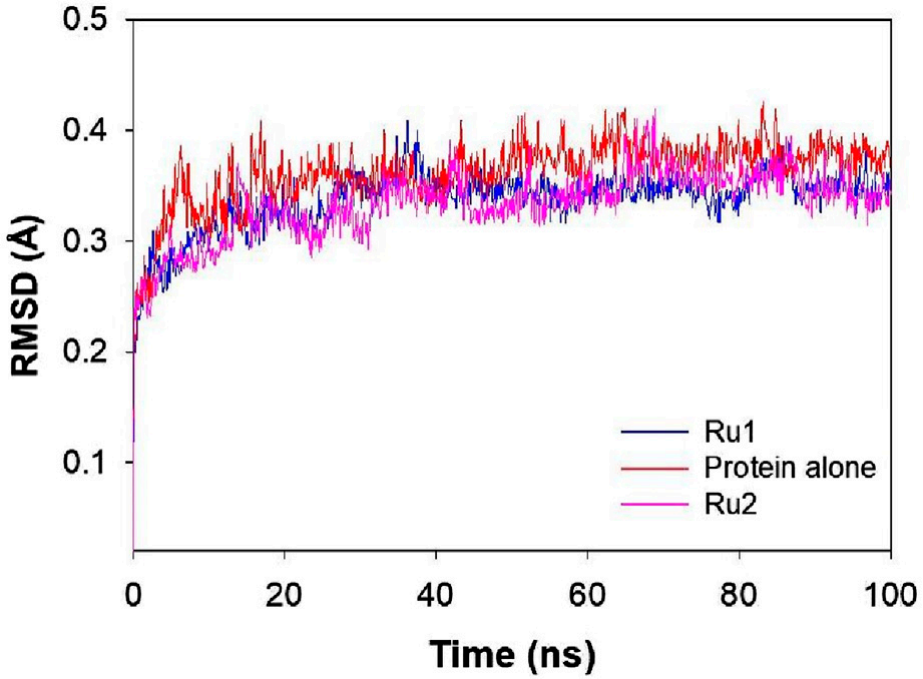
Plot depicting the RMSD of 4WOY in the presence and absence of **Ru1** and **Ru2** during the simulation period of 100 ns.

**FIGURE 9:**
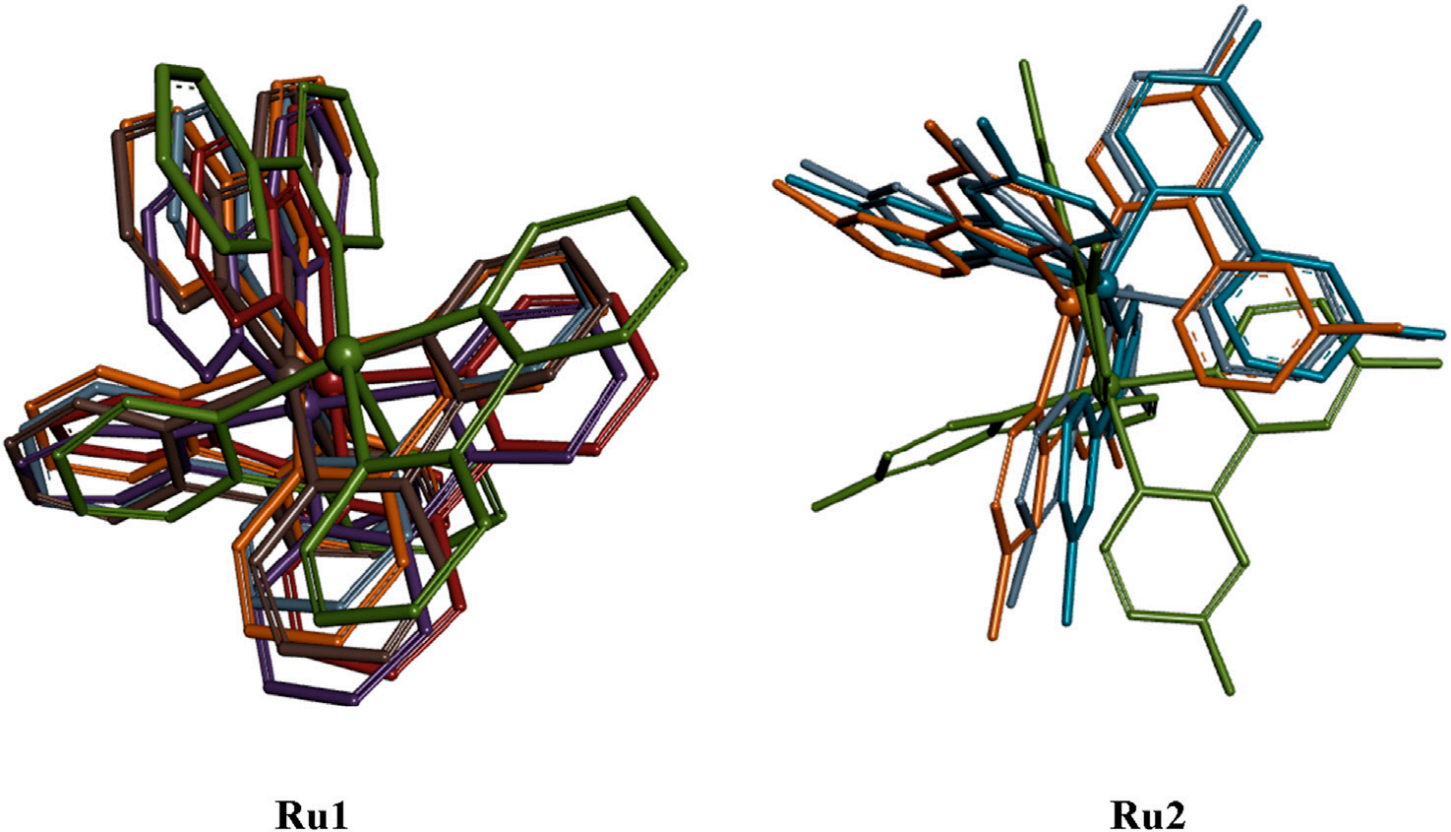
Overlaid structural geometries of **Ru1** and **Ru2** complexes (hydrogen excluded) during their interaction with the 4WOY protein at different molecular dynamic simulation periods (0–100 ns).

Figure 8 shows that a stable trajectory with RMSD of 3.37 ± 1.13 Å and 3.34 ± 0.99 Å was observed for **Ru1** and **Ru2**, respectively, while for the protein alone in the absence of any ligands, it was 3.62 ± 1.09 Å over a period of 100 ns. The deviation observed in the initial 20 ns was considered the stabilization period and was not considered in the process of evaluation. The results indicate that the interaction of **Ru1** and **Ru2** with the binding residues of 4WOY has negligible influence on the structural deformation of the protein. Overlaid ligand positions for **Ru1** and **Ru2** during the simulation period are shown in Figure 9.

#### Influence of the interaction of ruthenium complexes on RMSF trajectories

**FIGURE 10:**
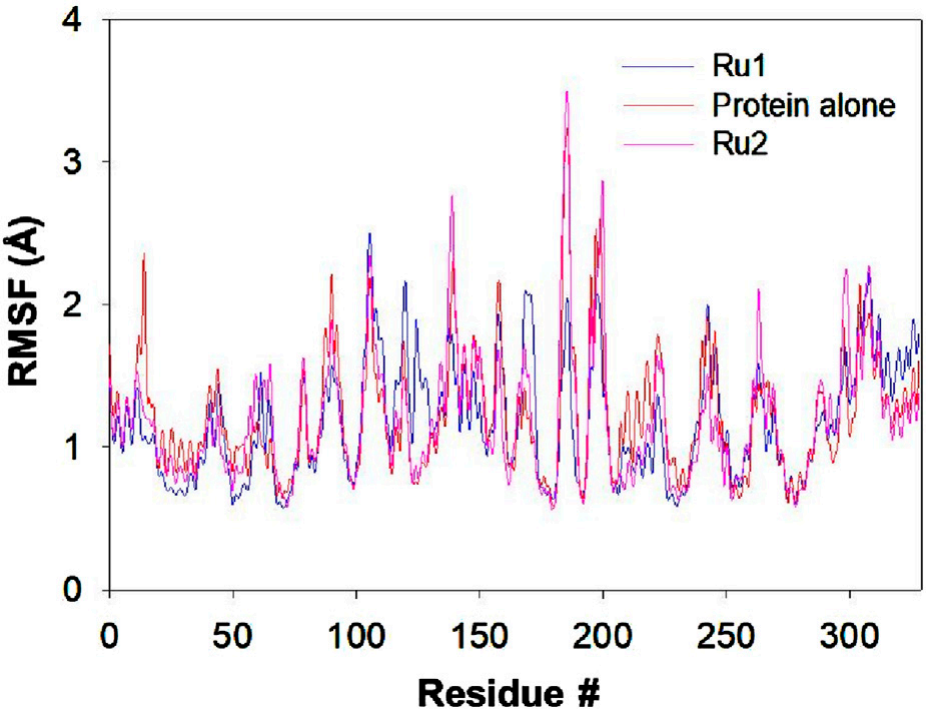
Plot depicting the RMSF trajectory for the 4WOY protein during its interaction with **Ru1** and **Ru2** for a simulation period of 100 ns.

The interaction of tris(polypyridyl)ruthenium(II) complexes with the 4WOY protein induces fluctuations of amino acid residues, which are evaluated by following the RMSF trajectories. The positional fluctuations were monitored for the amino acid residues upon the approach of **Ru1** and **Ru2**, and respective trajectories are depicted in Figure 10.

The residues E231, F232, E434, G246, E433, E295, C296, G324, R432, and L297 of 4WOY revealed RMSF >2 Å, during its interaction with **Ru1**, while in the case of **Ru2**, the residues S311, T312, A310, A326, S265, and L325 were revealed to possess values >2.5 Å. In the absence of any ligand, the residues S311, T312, A310, L325, and E323 showed fluctuations >2.5 Å. The results indicate that the observed deviations in the RMSF trajectories were significant in the case of **Ru1**, while no significant changes were observed for **Ru2**. The average deviation in RMSF for the protein in the absence of **Ru1** or **Ru2** is 1.22 ± 1.28 Å, while it is 1.17 ± 0.93 Å and 1.20 ± 1.44 Å in the presence of **Ru1** and **Ru2** respectively.

#### Influence of the interaction of ruthenium complexes on Rg trajectories

**FIGURE 11:**
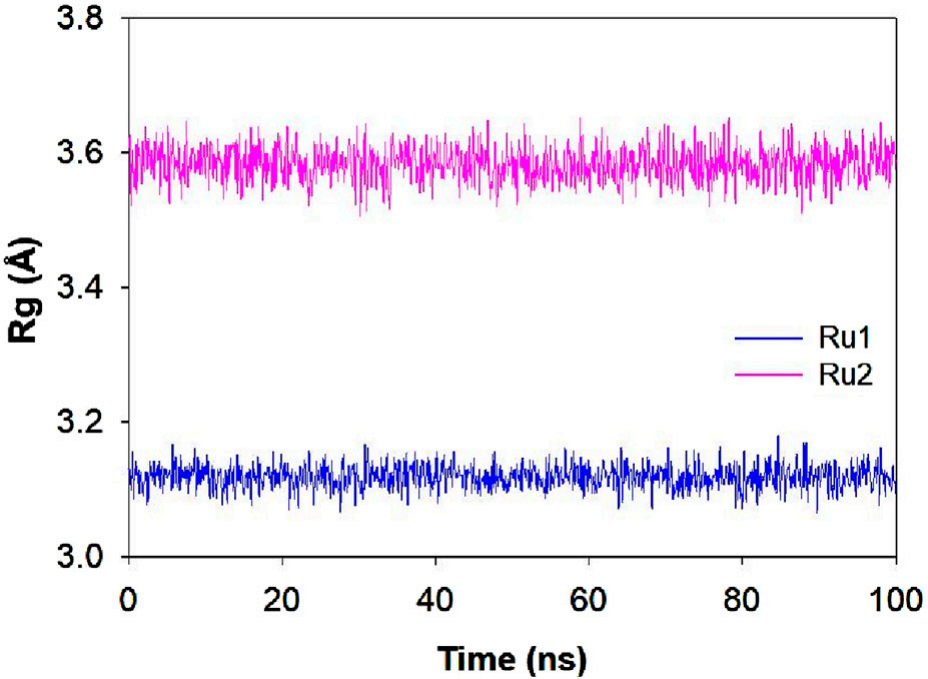
Plot depicting the Rg trajectory for **Ru1** and **Ru2** during their interaction with 4WOY for a simulation period of 100 ns.

The variation in the structural compactness of the protein upon the approach of the ligands (**Ru1** and **Ru2**) was monitored by following the radius of gyration during the simulation period of 100 ns. Rg indicates the positional variations of the ligand’s constituting atoms from the central axis of the ligand during its rotation when maximum energy was transferred. The average deviations observed in the Rg trajectories for **Ru1** and **Ru2** were 3.12 ± 0.08 Å and 3.58 ± 0.07 Å, respectively. The results indicate that the changes induced in the ligands were insignificant during its binding to 4WOY. The respective trajectories are depicted in Figure 11.

#### Influence of the interaction of ruthenium complexes on solvent-accessible surface area trajectories

**FIGURE 12:**
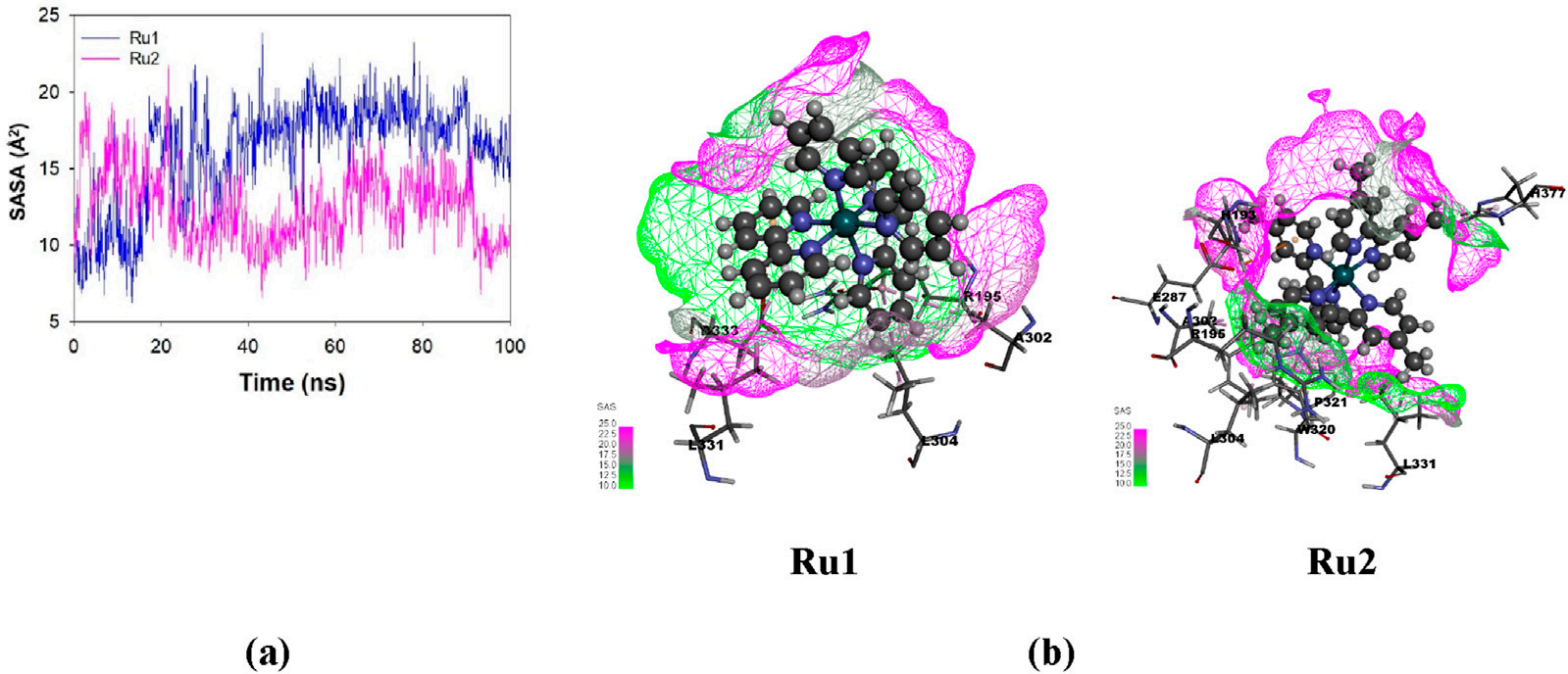
**(A)** Plot depicting the variation in solvent-accessible surface area (SASA) for **Ru1** and **Ru2** during their interaction with 4WOY for a simulation period of 100 ns. **(B)** Contour map depicting the solvent-accessible surface area for **Ru1** and **Ru2**.

The exposure of the ligand’s surface to the solvent environment is greatly influenced by its binding orientation during its interaction with 4WOY. The investigation of the solvent-accessible surface area will help to identify the key binding pocket residues on the protein target and, hence, the nature of interaction during the simulation period. The average fluctuations observed in the case **Ru1** (161.17 ± 87.21 Å^2^) are greater than **Ru2** (124.67 ± 75.30 Å^2^), indicating that the former is more exposed to solvents. The respective trajectories and contour surfaces are depicted in Figure 12.

The results revealed that the anticancer activity of **Ru1** complex is higher than that of **Ru2**. In **Ru2**, the electron-releasing methyl groups present in the dmbpy ligand are believed to alter the physical and chemical properties of the complex. These changes might significantly influence the complex toward interacting with reactive oxygen species (ROS) or other radicals in biological systems (Basudeb Saha and Stanbury, 2000). Thus, it was revealed that the anticancer activity of these complexes depends on the nature of the ligands present in these metal complexes.

### Evaluation of antioxidant potential

The interaction potential of these complexes with quinones has shown promising results for charge transfer mediating potential. Because it is well known that tris(polypyridyl)ruthenium(II) complexes can undergo charge transfer reactions, it will be interesting to investigate their antioxidant potential. Herein, the 1,1-diphenyl-2-picrylhydrazyl (DPPH) free radical scavenging assay was followed to assess antioxidant activity. The antioxidant activity of **Ru2** was measured following the previously reported method with slight modifications and was compared with **Ru1**. The DPPH radical is known for its radical scavenging ability. Free radical scavenging activity was expressed as percentage inhibition and was calculated using the formula (A_0_ − A_1_)/A_0_ × 100, where A_0_ is the control absorbance and A_1_ is the sample absorbance. All investigations were performed in triplicate, and antioxidant activity was quantified. The DPPH scavenging activity was calculated using the equation:

$$\text{DPPH scavenging activity }\left(\%\right)=\left(C\;–\;S\right)\;/C\times 100,$$ (6)

where C denotes the control sample, and S denotes the test samples (**Ru1** and **Ru2**).

**FIGURE 13:**
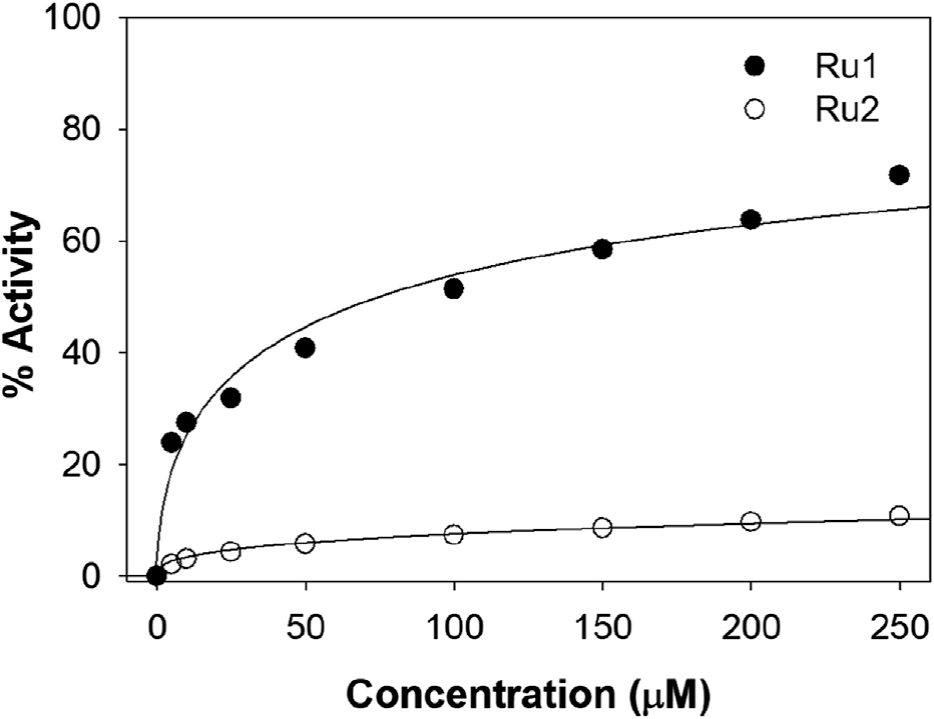
Plot depicting the DPPH antioxidant potential at various concentrations of **Ru1** and **Ru2** complexes.

Figure 13 depicts the percentage radical scavenging activity of **Ru1** and **Ru2** in the concentration range of 0–250 µM. It was revealed that the scavenging ability for **Ru1** is higher than **Ru2** at all concentrations followed in the study. The activities at 100 µM of **Ru1** and **Ru2** were 51.3% and 7.3%, respectively. The above investigations reveal that the electronic and structural features were more favorable for **Ru1** to act as an antioxidant than **Ru2**. The presence of an electron-donating methyl group in the dmbpy ligand decreases the antioxidant activity of the **Ru2** complex. The methyl groups present in the dmbpy ligand donate the electron density to the aromatic ring, making the **Ru2** complex less effective for stabilizing the free radicals, which might even reduce its ability to scavenge free radicals (Basudeb Saha and Stanbury, 2000).

### Evaluation of antimicrobial activity

**FIGURE 14:**
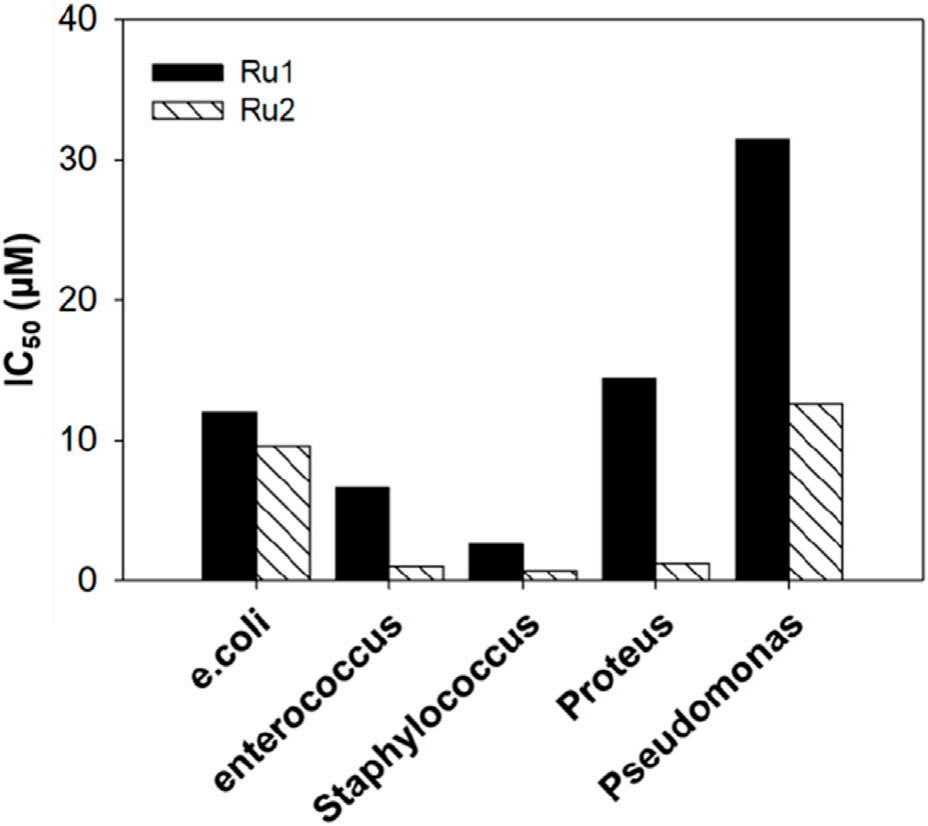
Bar graph depicting the antimicrobial activity of tris(polypyridyl)ruthenium(II) complexes.

To further investigate the broad-scope biological applications of these ruthenium complexes, the antibacterial activity was determined by following the conventional liquid turbidity method. For this purpose, three Gram-positive bacteria—*Enterococcus, Staphylococcus*, and *Pseudomonas*—and two Gram-negative bacteria—*E. coli and Proteus*—were chosen. Here, the overnight grown bacterial cultures were diluted 1,000 fold in the presence of various concentrations (0–500 µM) of **Ru1** and **Ru2** and further incubated in a shaker incubator at 37°C for a period of 8 h. The inhibition of growth was followed by monitoring the OD at 600 nm. The IC_50_ values were evaluated by plotting the optical density against various concentrations, and 50% of the maximum inhibitory concentrations (IC_50_) are given in Table 7. Table 7 shows that the antimicrobial activity of **Ru2** was better than **Ru1**. The bar graph in Figure 14 depicts the variations in the IC_50_ values for various gram positive and gram negative bacteria.

**TABLE 7:** IC_50_ values depicting the antimicrobial activity of the tris(polypyridyl)ruthenium(II) complexes.

Bacteria	IC_50_ (µM)		
	Ru1	Ru2	Ciprofloxacin
*Escherichia coli*	12.00	9.62	0.21
*Enterococcus* *faecium*	6.64	1.06	0.49
*Staphylococcus aureus*	2.55	0.73	3.27
*Proteus* *vulgaris*	14.4	1.24	0.98
*Pseudomonas aeruginosa*	31.4	12.6	0.24

## Conclusion

Herein, the broad-scope applications of tris(polypyridyl) ruthenium(II) complexes were investigated. The ground state and excited state photophysical properties of these complexes were evaluated from their electronic absorption and fluorescence emission spectra. A prominent blue shift was observed in the absorption spectrum. A red shift was observed in the emission spectrum of the ruthenium complex with a bpy ligand, while a blue shift was observed for the ruthenium complex with a dmbpy ligand. The long wavelength absorption band observed in the spectrum could be assigned to the MLCT transition. Evaluations on the influence of various quinones on the electron transfer properties revealed the potential of these complexes to be involved in charge transfer interactions. The same was revealed from the calculated binding constant values. The change in dipole moment upon excitation to higher energy levels was insignificant. The HOMO and LUMO charge density profiles support the same interpretation. The anticancer potential of these complexes was evaluated through docking investigations. The binding site is mostly composed of hydrophilic and hydrophobic residues, and the interactions were stabilized by π-alkyl interactions. The molecular dynamic simulations have revealed a comparable anticancer activity for **Ru2** when targeting the 4WOY protein compared to **Ru1**. The antioxidant activity of **Ru2** is slightly lower than that of **Ru1**, and the antimicrobial activity is higher for **Ru1**. The photophysical and biological activities of these synthesized complexes depend on the nature of the ligands present in the complexes.

## Data Availability

The original contributions presented in the study are included in the article/Supplementary Material; further inquiries can be directed to the corresponding author.
