# Supplementary material for: Investigation of photophysical properties and potential biological applications of substituted tris(polypyridyl)ruthenium(II) complexes
Source: Front Chem. 2025 Feb 3;13:1491598. doi: 10.3389/fchem.2025.1491598 (PMC11830695; doi:10.3389/fchem.2025.1491598)
Supplement: Supplementary file 1 [file Table1.docx]

**SUPPORTING INFORMATION**

**Investigation of photophysical properties and potential biological applications of substituted Tris(polypyridyl)ruthenium(II) complexes**

^1^T.Sumitha Celin, ^1^G.Allen Gnana Raj, ^2^T. S. Prathima, ^2^M. M. Balamurali*

^1^Department of Chemistry & Research Centre, Scott Christian College (Autonomous), Nagercoil, Tamilnadu, India

^2^Department of Chemistry, School of Advanced Sciences, Vellore Institute of Technology, Chennai, Tamilnadu, India

Email: [allengraj@gmail.com](mailto:allengraj@gmail.com), [balamurali.mm@vit.ac.in](mailto:balamurali.mm@vit.ac.in)

**Synthesis of Ru(II)-polypyridine complexes**

*Tris (2,2'- bipyridine) Ruthenium (II) Chloride, [Ru(bpy)_3_](BF_4_)_2_*

0.5 g of RuCl_3_.3H_2_O was reacted with 0.6 g of 2, 2ˈ- bipyridine in 25 ml of ethanol. The above solution was refluxed for 20 hours for an orange red complex to form. Following this a saturated solution of sodium tetrafluoroborate was then added dropwise until an orange precipitate is formed. Further purification of the above obtained crude product was performed in a silica gel column employing n-propanol as the eluent. The pure complex was obtained upon subsequent evaporation of the solvent. The authenticity of the complex was done by monitoring the absorption maximum (λ_abs_^max^) and emission maximum (λ_em_^max^) of the complex in CH_3_CN as 448 nm and 596 nm respectively. The values were in agreement with the reported values.

^1^H-NMR (DMSO-d_6_): 8.85-8.91(d,2H), 8.16(d,2H), 7.72(t,2H), 7.53(t,2H).

IR (KBr, cm^-1^): 3068 (C-H), 1617(C=C), 1476(C-C), 1155(C=N) and 808(Ru-N) υ/cm^-1^.

*Tris(4,4′-dimethyl-2,2′-bipyridine)ruthenium(II)tetrafluoroborate, [Ru(dmbpy)_3_](BF_4_)_2_.*

RuCl_3_.3H_2_O (1 mM) and 4,4ˈ-dimethyl-2,2ˈ-bipyridine (3 mM) were dissolved in 20 ml of ethylene glycol and refluxed for 4 hours. The solution was then allowed to cool at room temperature and filtered to remove any insoluble impurities. A saturated solution of sodium tetrafluoroborate was then added dropwise into the filtrate until an orange precipitate is formed. The product was filtered, washed with cold water and diethyl ether and further dried in a vacuum desiccator. The product was further purified by recrystallisation from water. The absorption maximum (λ_abs_^max^) and emission maximum (λ_em_^max^) of the complex in CH_3_CN are 458 nm and 601 nm respectively.

^1^H-NMR (DMSO-d_6_): 8.68(d, 2H), 7.54-7.55(t,2H) and 7.34-7.35(t,2H) and 2.5 (6H (CH_3_),s).

IR (KBr, cm^-1^): 1068(C-N), 1505(C=C),2345(C-H) and 2966(*sp*^3^-C-H) υ/cm^-1^.

1. Kalaiyar Swarnalatha, Eswaran Rajkumar, S, Rajagopal, R, Ramaraj, I, Sadhiya Banu & Perumal Ramamurthy, 2011, ‘Proton coupled ET reaction of phenols with excited state ruthenium(II) Polypyridyl complexes’, Journal of Physical Organic Chemistry, vol. 24, no.1, pp. 14 - 21.
2. Saha, B & Stanbury, DM 2000, ‘Thermal and photochemical reduction of aqueous chlorine by ruthenium(II) polypyridyl complexes’, Inorganic Chemistry, vol. 39, pp. 1294 -1300.

**Table 1.** Geometry optimized coordinates of tris(polypyridyl)ruthenium(II) complexes.

| **Ru1** | \| C \| -0.6751 \| -4.2068 \| -2.4246 \| \| --- \| --- \| --- \| --- \| \| C \| 0.3302 \| -3.3064 \| -2.8853 \| \| C \| 0.5278 \| -2.1142 \| -2.1884 \| \| N \| -0.1906 \| -1.7678 \| -1.0819 \| \| C \| -1.1595 \| -2.6570 \| -0.5968 \| \| C \| -1.4083 \| -3.8778 \| -1.2893 \| \| C \| -1.8435 \| -2.2366 \| 0.5987 \| \| N \| -1.4884 \| -0.9702 \| 1.0830 \| \| C \| -2.1221 \| -0.4853 \| 2.1892 \| \| C \| -3.0962 \| -1.2002 \| 2.8864 \| \| C \| -3.4450 \| -2.5042 \| 2.4264 \| \| C \| -2.8201 \| -3.0101 \| 1.2914 \| \| C \| -0.4515 \| 4.2359 \| 2.4252 \| \| C \| 0.5052 \| 3.2836 \| 2.8853 \| \| C \| 0.6394 \| 2.0828 \| 2.1882 \| \| N \| -0.0966 \| 1.7751 \| 1.0817 \| \| C \| -1.0173 \| 2.7142 \| 0.5970 \| \| C \| -1.2013 \| 3.9463 \| 1.2900 \| \| C \| -1.7226 \| 2.3309 \| -0.5985 \| \| N \| -1.4347 \| 1.0478 \| -1.0836 \| \| C \| -2.0930 \| 0.5976 \| -2.1900 \| \| C \| -3.0282 \| 1.3632 \| -2.8867 \| \| C \| -3.3078 \| 2.6835 \| -2.4260 \| \| C \| -2.6570 \| 3.1552 \| -1.2908 \| \| C \| 3.9802 \| 1.5216 \| -2.4252 \| \| C \| 2.6974 \| 1.9426 \| -2.8842 \| \| C \| 1.5667 \| 1.5160 \| -2.1874 \| \| N \| 1.6270 \| 0.7188 \| -1.0824 \| \| C \| 2.8819 \| 0.3251 \| -0.5979 \| \| C \| 4.0630 \| 0.7208 \| -1.2908 \| \| C \| 2.8605 \| -0.4771 \| 0.5979 \| \| N \| 1.5865 \| -0.8037 \| 1.0823 \| \| C \| 1.4839 \| -1.5963 \| 2.1875 \| \| C \| 2.5903 \| -2.0820 \| 2.8846 \| \| C \| 3.8937 \| -1.7298 \| 2.4256 \| \| C \| 4.0189 \| -0.9348 \| 1.2911 \| \| Ru \| 0.0011 \| -0.0001 \| -0.0003 \| \| H \| 1.3463 \| 1.3266 \| 2.5093 \| \| H \| 1.1237 \| 3.4751 \| 3.7559 \| \| H \| -1.9352 \| 4.6592 \| 0.9281 \| \| H \| -2.8564 \| 4.1587 \| -0.9287 \| \| H \| -3.5255 \| 0.9483 \| -3.7572 \| \| H \| -1.8425 \| -0.4069 \| -2.5111 \| \| H \| -3.5707 \| -0.7592 \| 3.7568 \| \| H \| -1.8188 \| 0.5046 \| 2.5098 \| \| H \| 0.4749 \| -1.8303 \| 2.5070 \| \| H \| 2.4461 \| -2.7144 \| 3.7543 \| \| H \| 5.0036 \| -0.6557 \| 0.9300 \| \| H \| 0.9375 \| -3.5304 \| -3.7561 \| \| H \| 1.2734 \| -1.3962 \| -2.5099 \| \| H \| 0.5716 \| 1.8032 \| -2.5068 \| \| H \| 2.5869 \| 2.5821 \| -3.7537 \| \| H \| -3.0721 \| -4.0018 \| 0.9297 \| \| H \| -2.1786 \| -4.5511 \| -0.9271 \| \| H \| 5.0315 \| 0.3899 \| -0.9297 \| \| H \| 4.8811 \| 1.8217 \| -2.9537 \| \| H \| 4.7774 \| -2.0772 \| 2.9542 \| \| H \| -0.5934 \| 5.1749 \| 2.9536 \| \| H \| -4.0192 \| 3.3130 \| -2.9539 \| \| H \| -4.1885 \| -3.0950 \| 2.9547 \| \| H \| -0.8665 \| -5.1372 \| -2.9527 \| | **Ru2** | \| C \| 3.7200 \| 2.1273 \| -2.4241 \| \| --- \| --- \| --- \| --- \| \| C \| 2.3766 \| 2.3367 \| -2.8724 \| \| C \| 1.3231 \| 1.7412 \| -2.1812 \| \| N \| 1.4987 \| 0.9601 \| -1.0787 \| \| C \| 2.7978 \| 0.7643 \| -0.5967 \| \| C \| 3.9047 \| 1.3410 \| -1.2891 \| \| C \| 2.9003 \| -0.0351 \| 0.5971 \| \| N \| 1.6925 \| -0.5521 \| 1.0795 \| \| C \| 1.7202 \| -1.3530 \| 2.1815 \| \| C \| 2.8902 \| -1.6640 \| 2.8718 \| \| C \| 4.1373 \| -1.1224 \| 2.4235 \| \| C \| 4.1171 \| -0.3144 \| 1.2887 \| \| C \| -3.0401 \| -3.0196 \| 2.4245 \| \| C \| -2.8848 \| -1.6689 \| 2.8727 \| \| C \| -2.0302 \| -0.8118 \| 2.1818 \| \| N \| -1.3237 \| -1.1889 \| 1.0795 \| \| C \| -1.4806 \| -2.4932 \| 0.5972 \| \| C \| -2.3311 \| -3.4068 \| 1.2895 \| \| C \| -0.7375 \| -2.8046 \| -0.5966 \| \| N \| 0.0820 \| -1.7778 \| -1.0791 \| \| C \| 0.8460 \| -2.0172 \| -2.1818 \| \| C \| 0.8340 \| -3.2276 \| -2.8724 \| \| C \| -0.0198 \| -4.2857 \| -2.4239 \| \| C \| -0.7926 \| -4.0516 \| -1.2887 \| \| C \| -3.7009 \| 2.1584 \| -2.4252 \| \| C \| -3.2108 \| 0.8900 \| -2.8733 \| \| C \| -2.1686 \| 0.2752 \| -2.1819 \| \| N \| -1.5799 \| 0.8177 \| -1.0794 \| \| C \| -2.0598 \| 2.0408 \| -0.5974 \| \| C \| -3.1122 \| 2.7114 \| -1.2901 \| \| C \| -1.4194 \| 2.5287 \| 0.5969 \| \| N \| -0.3682 \| 1.7409 \| 1.0795 \| \| C \| 0.3108 \| 2.1645 \| 2.1824 \| \| C \| -0.0052 \| 3.3328 \| 2.8733 \| \| C \| -1.0975 \| 4.1423 \| 2.4246 \| \| C \| -1.7865 \| 3.7216 \| 1.2892 \| \| Ru \| 0.0003 \| 0.0000 \| 0.0003 \| \| H \| -1.8863 \| 0.2130 \| 2.5047 \| \| H \| -3.4250 \| -1.3034 \| 3.7412 \| \| H \| -2.4233 \| -4.4248 \| 0.9230 \| \| H \| -1.4535 \| -4.8313 \| -0.9220 \| \| H \| 1.4730 \| -3.3564 \| -3.7412 \| \| H \| 1.4758 \| -1.1964 \| -2.5053 \| \| H \| 2.8444 \| -2.3152 \| 3.7399 \| \| H \| 0.7614 \| -1.7424 \| 2.5044 \| \| H \| 1.1266 \| 1.5280 \| 2.5056 \| \| H \| 0.5808 \| 3.6180 \| 3.7422 \| \| H \| -2.6221 \| 4.3103 \| 0.9225 \| \| H \| 2.1679 \| 2.9544 \| -3.7411 \| \| H \| 0.2969 \| 1.8754 \| -2.5041 \| \| H \| -1.7719 \| -0.6807 \| -2.5043 \| \| H \| -3.6414 \| 0.4005 \| -3.7419 \| \| H \| 5.0445 \| 0.1152 \| 0.9220 \| \| H \| 4.9106 \| 1.1591 \| -0.9227 \| \| H \| -3.4574 \| 3.6736 \| -0.9238 \| \| C \| -0.0649 \| -5.6023 \| -3.1701 \| \| H \| 0.9277 \| -6.0740 \| -3.1981 \| \| H \| -0.7607 \| -6.3077 \| -2.7019 \| \| H \| -0.3811 \| -5.4568 \| -4.2129 \| \| C \| -3.9475 \| -3.9749 \| 3.1704 \| \| H \| -4.9797 \| -3.5978 \| 3.1984 \| \| H \| -3.9628 \| -4.9656 \| 2.7021 \| \| H \| -3.6221 \| -4.0985 \| 4.2133 \| \| C \| 4.8821 \| -2.7471 \| 3.1709 \| \| H \| 4.7924 \| 3.8424 \| -3.2013 \| \| H \| 5.8413 \| 2.5000 \| -2.7017 \| \| H \| 4.9154 \| 2.3984 \| -4.2130 \| \| C \| 5.4187 \| -1.4299 \| 3.1690 \| \| H \| 5.6110 \| -2.5120 \| 3.1934 \| \| H \| 6.2833 \| -0.9440 \| 2.7027 \| \| H \| 5.3618 \| -1.0899 \| 4.2129 \| \| C \| -4.8190 \| 2.8549 \| -3.1716 \| \| H \| -5.7232 \| 2.2302 \| -3.2000 \| \| H \| -5.0832 \| 3.8099 \| -2.7034 \| \| H \| -4.5347 \| 3.0564 \| -4.2143 \| \| C \| -1.4723 \| 5.4051 \| 3.1708 \| \| H \| -0.6300 \| 6.1110 \| 3.1997 \| \| H \| -2.3226 \| 5.9134 \| 2.7022 \| \| H \| -1.7425 \| 5.1846 \| 4.2134 \| |
| --- | --- | --- | --- | --- | --- | --- | --- | --- | --- | --- | --- | --- | --- | --- | --- | --- | --- | --- | --- | --- | --- | --- | --- | --- | --- | --- | --- | --- | --- | --- | --- | --- | --- | --- | --- | --- | --- | --- | --- | --- | --- | --- | --- | --- | --- | --- | --- | --- | --- | --- | --- | --- | --- | --- | --- | --- | --- | --- | --- | --- | --- | --- | --- | --- | --- | --- | --- | --- | --- | --- | --- | --- | --- | --- | --- | --- | --- | --- | --- | --- | --- | --- | --- | --- | --- | --- | --- | --- | --- | --- | --- | --- | --- | --- | --- | --- | --- | --- | --- | --- | --- | --- | --- | --- | --- | --- | --- | --- | --- | --- | --- | --- | --- | --- | --- | --- | --- | --- | --- | --- | --- | --- | --- | --- | --- | --- | --- | --- | --- | --- | --- | --- | --- | --- | --- | --- | --- | --- | --- | --- | --- | --- | --- | --- | --- | --- | --- | --- | --- | --- | --- | --- | --- | --- | --- | --- | --- | --- | --- | --- | --- | --- | --- | --- | --- | --- | --- | --- | --- | --- | --- | --- | --- | --- | --- | --- | --- | --- | --- | --- | --- | --- | --- | --- | --- | --- | --- | --- | --- | --- | --- | --- | --- | --- | --- | --- | --- | --- | --- | --- | --- | --- | --- | --- | --- | --- | --- | --- | --- | --- | --- | --- | --- | --- | --- | --- | --- | --- | --- | --- | --- | --- | --- | --- | --- | --- | --- | --- | --- | --- | --- | --- | --- | --- | --- | --- | --- | --- | --- | --- | --- | --- | --- | --- | --- | --- | --- | --- | --- | --- | --- | --- | --- | --- | --- | --- | --- | --- | --- | --- | --- | --- | --- | --- | --- | --- | --- | --- | --- | --- | --- | --- | --- | --- | --- | --- | --- | --- | --- | --- | --- | --- | --- | --- | --- | --- | --- | --- | --- | --- | --- | --- | --- | --- | --- | --- | --- | --- | --- | --- | --- | --- | --- | --- | --- | --- | --- | --- | --- | --- | --- | --- | --- | --- | --- | --- | --- | --- | --- | --- | --- | --- | --- | --- | --- | --- | --- | --- | --- | --- | --- | --- | --- | --- | --- | --- | --- | --- | --- | --- | --- | --- | --- | --- | --- | --- | --- | --- | --- | --- | --- | --- | --- | --- | --- | --- | --- | --- | --- | --- | --- | --- | --- | --- | --- | --- | --- | --- | --- | --- | --- | --- | --- | --- | --- | --- | --- | --- | --- | --- | --- | --- | --- | --- | --- | --- | --- | --- | --- | --- | --- | --- | --- | --- | --- | --- | --- | --- | --- | --- | --- | --- | --- | --- | --- | --- | --- | --- | --- | --- | --- | --- | --- | --- | --- | --- | --- | --- | --- | --- | --- | --- | --- | --- | --- | --- | --- | --- | --- | --- | --- | --- | --- | --- | --- | --- | --- | --- | --- | --- | --- | --- | --- | --- | --- | --- | --- | --- | --- | --- | --- | --- | --- | --- | --- | --- | --- | --- | --- | --- | --- | --- | --- | --- | --- | --- | --- | --- | --- | --- | --- | --- | --- | --- | --- | --- | --- | --- | --- | --- | --- | --- | --- | --- | --- | --- | --- | --- | --- | --- | --- | --- | --- | --- | --- | --- | --- | --- | --- | --- | --- | --- | --- | --- | --- | --- | --- | --- | --- | --- | --- | --- | --- | --- | --- | --- | --- | --- | --- | --- | --- | --- | --- | --- | --- | --- | --- | --- | --- | --- | --- | --- | --- | --- | --- | --- | --- | --- | --- | --- | --- | --- | --- | --- | --- | --- | --- | --- | --- | --- | --- | --- | --- | --- | --- | --- | --- | --- | --- | --- | --- | --- | --- |
